# Identification of sulfakinin receptors (SKR) in *Tenebrio molitor* beetle and the influence of sulfakinins on carbohydrates metabolism

**DOI:** 10.1007/s00360-020-01300-6

**Published:** 2020-08-04

**Authors:** M. Słocińska, S. Chowański, P. Marciniak

**Affiliations:** Department of Animal Physiology and Development, Faculty of Biology, Adam Mickiewicz University, Poznań, ul. Uniwersytetu Poznańskiego 6, 61-614 Poznan, Poland

**Keywords:** Sulfated, Nonsulfated sulfakinin, G protein-coupled receptors, *T. molitor*, Insect

## Abstract

Sulfakinins (SKs) are pleiotropic neuropeptides commonly found in insects, structurally and functionally homologous to the mammalian gastrin/cholecystokinin (CCK) neuropeptides. SKs together with sulfakinin receptors (SKRs) are involved in sulfakinin signaling responsible for variety of biological functions, including food intake or fatty acid metabolism. In the present study, we determined the distribution of SKRs in *Tenebrio molitor* larvae and characterized the impact of nonsulfated and sulfated SKs on carbohydrates and insulin-like peptides (ILPs) level in beetle hemolymph. Our results indicate the presence of both sulfakinin receptors, SKR1 and SKR2, in the nervous system of *T. molitor.* The distribution of SKR2 in peripheral tissues was more widespread than SKR1, and their transcripts have been found in fat body, gut and hemolymph. This is also the first evidence for SKRs presence in insect hemocytes indicating immunotropic activity of SKs. Moreover, in the present study, we have demonstrated that SKs regulate ILPs and carbohydrates level in insect hemolymph, and that sulfation is not crucial for peptides activity. Our study confirms the role of SKs in maintaining energy homeostasis in beetles.

## Introduction

Insect SKs are a family of neuropeptides homologous to mammalian gastrin/cholecystokinin (CCK). Sulfated sulfakinins (sSK) contain a sulfated tyrosine residue in their conserved C-terminal heptapeptide (DY(SO_3_)GHM/LRFamide), although nonsulfated SKs (nSK) with biological activity are found in vivo as well (Nichols et al. 2008; Słocińska et al. 2015b; Marciniak et al. 2011; Adamski et al. 2019).

The first sSKs with myotropic activity on the isolated cockroach hindgut were isolated from head extracts of cockroach *Leucophaea maderae* and then widely identified in a variety of insect species (Nichols et al. 1988; Schoofs et al. 1990; Duve et al. 1995; Predel et al. 1999; Veenstra 1989; Maestro et al. 2001) with activities in diverse of biological processes including food intake regulation (Zels et al. 2015; Yu et al. 2013; Al-Alkawi et al. 2017; Downer et al. 2007), release of digestive enzymes (Nachman et al. 1997; Harshini et al. 2002; Zels et al. 2015) (Nachman et al. 1997), modulation of odor preferences (Nichols et al. 2008), locomotion (Nichols et al. 2008; Chen et al. 2012), synaptic growth (Chen and Ganetzky 2012) or aggression (Williams et al. 2014). Nonsulfated sulfakinins act as a myotropic peptides on visceral muscles, including heart (Marciniak et al. 2011; Nichols et al. 2009), oviduct and ejaculatory duct (Marciniak et al. 2011) and furthermore, are engaged in the regulation of carbohydrates and lipids metabolism (Słocińska et al. 2019, 2015a, b, 2016). As was shown, SKs are co-localized with insulin-like peptides (ILPs) in insulin-producing cells (IPCs) in the larval and adult brains of *Drosophila melanogaster* (Söderberg et al. 2012; Nassel et al. 2013). Lin et al. (2016) reported that interplay of insulin and sulfakinin signaling plays a role in the regulation of food intake in larval stages of *Tribolium castaneum.*

Sulfakinin signaling involves sulfakinin peptides (SKs) and sulfakinin receptors (SKRs), which similarly to cholecystokinin receptors belong to the family of G-protein-coupled receptors (GPCRs). Based on the sequence similarity to cholecystokinin receptors (CCKRs), two SKR genes named DSKR1 and DSKR2 have been identified in *D. melanogaster* (Hauser et al. 2006; Kubiak et al. 2002). Two next SKR genes were identified in *Periplaneta americana* (Wicher et al. 2007) and *T. castaneum* (Hauser et al. 2008) and the receptors were functionally characterized (Yu et al. 2013; Yu and Smagghe 2014b; Zels et al. 2014). The transcript distribution for both receptors in this species was shown to be stage and tissue specific with the predominant expression in the nervous system (Zels et al. 2014; Yu and Smagghe 2014b; Yu et al. 2013). Recently, it was also confirmed in *R. prolixus* (Bloom et al. 2019). In *T. castaneum,* silencing of two TcSKR genes resulted in altered feeding behavior to different extent with again strong evidence that TcSKRs are involved in the regulation of food intake (Yu et al. 2013; Yu and Smagghe 2014a, b).

It was exhibited that sSK peptide is 1000–10,000 times more potent than nsSK to activate both SKR1 and SKR2 in *T. castaneum* (Yu and Smagghe 2014b). Moreover, in hemimetabolous insect, *Locusta migratoria*, sulfation of the tyrosine residue was crucial for the observed effects on digestive enzyme secretion (Zels et al. 2015), and in *R. prolixus* indispensable for binding SKs to the SKRs (Bloom et al. 2019). In *Tenebrio molitor*, sulfation causes different changes in fatty acid profile in fat body tissue (Słocińska et al. 2019). Yu et al. (2015) showed that sulfate moiety on tyrosyl residue contributed to the higher activity of sSK over nsSK by enhancing the binding of sulfakinin peptide to the receptor.

In this study, we analyzed the effect of SKs on carbohydrate profile and insulin-like peptides (ILPs) level in *T. molitor* hemolymph. Based on the present and previously reported data (Słocińska et al. 2016), we hypothesize that insulin signaling crosstalks with sulfakinin pathways to maintain carbohydrates homeostasis. To check the importance of sulfated tyrosine residue in sugar metabolism, sulfated and nonsulfated SKs were applied. Furthermore, to prove whether the effects are based upon direct action via the receptor, we performed reverse transcriptase PCR (RT-PCR) analysis to know the distribution of transcripts encoding SKR1–Tenmo-SKR1 and SKR2–Tenmo-SKR2 in different tissues of *T. molitor* larvae.

## Materials and methods

### Insects

Larvae of the *T. molitor* beetle were reared under laboratory conditions at a temperature of 28 °C and a relative humidity of 65–70%. Food, including lettuce, carrots, powdered milk and eggs, as well as water, were provided ad libitum. A stock culture of *T. molitor* was maintained at the Department of Animal Physiology and Development at Adam Mickiewicz University in Poznań. All the experiments were performed on the same batch of insect culture. Only feeding larvae from 15 to 16 instar, of approximately 110–140 mg of weight, were used for all the experimental variants.

### Transcriptome sequencing, database search and sequence comparison

Transcriptomic data from *T. molitor* were obtained after Illumina Hiseq sequencing of total RNA extracted from the brains and retrocerebral complexes of adult beetles performed at Beijing Genomics Institute (Shenzhen, China). After initial filtering of low-quality reads and adaptor removal, clean reads were de novo assembled using Trinity and used for local tblastn with receptor sequence from *T. castaneum* (AGK29938.1 for SKR1 and XP_972750.1 for SKR2) to find *T. molitor* receptors sequences. The transcriptome used was submitted to sequence read archive database (SRA) under the following accession numbers BioProject: PRJNA608239; SRR11184806 and SRR11358229.

The established protein sequence of the Tenmo-SKRs ORF was analyzed for the presence of putative transmembrane regions with the software programs PSIPRED–MEMSAT (https://bioinf.cs.ucl.ac.uk/psipred/) (Nugent and Jones 2009).

Protein sequence alignment of Tenmo-SKRs with other Coleopteran SKRs was performed with Clustal W (https://embnet.vital-it.ch/software/ClustalW.html) and includes for SKR1 *T. castaneum* (AGK29938.1), *Anoplophora glabripennis* (XP_018572599.1), *Ontophagus taurus* (XP_022903023.1), *Aethina tumida* (XP_019872041.1), *Nicrophorus vespilloides* (XP_017773250.1) and for SKR2 *T. castaneum* (XP_972750.1), *N. vespilloides* (XP_017773275.1)*, Rhynchophorus ferrugineus* (QGA72519.1), *Sitophilus oryzae* (XP_030750622.1), *Dendroctonus ponderosae* (XP_019756917.1), *A. glabripennis* (XP_018573812.1). All alignments and similarity analysis were visualized with the usage of Jalview and Ugene software.

### Receptor transcript distribution

Transcript profiles of Tenmo-SKR1 and Tenmo-SKR2 were determined by reverse transcriptase PCR (RT-PCR) in various tissues of *T. molitor* larvae. RT-PCR was performed according to a modification of the method described by Marone et al. (2001). Whole body or suitable tissues/organs (nervous system—brain and ventral nerve cord, fat body, gut and haemolymph) after dissection were transferred to 150 µL of RNA lysis buffer (Zymo Research, USA) and homogenized for 3 min using a pellet homogenizer. The homogenized tissues/organs were immediately frozen in liquid nitrogen and then stored at − 80 °C. A Quick-RNA^®^ Mini Prep kit (Zymo Research, USA) was used for RNA isolation. The RNA concentration was determined with a Synergy H1 Hybrid Multi-Mode Microplate Reader (BioTek, USA). Reverse transcription of the same amount of isolated RNA to cDNA was accomplished using the RevertAid™ Reverse Transcriptase kit (Thermo-Fisher Scientific, USA) according to the manufacturer’s protocol. PCR analyses were conducted using a T100™ Thermal Cycler (Bio-Rad, USA). The primers were designed based on the sequences of Tenmo-SKR1 and Tenmo-SKR2 using Primer3 software (Untergasser et al. 2012). The primer pair for Tenmo-SKR1 was created to amplify fragments of 129 bp with the following sequences Fw 5′-TATGTTATTATATTCGTGTTGTC-3′ and Rev 5′-GAGAAGTAAGTCGGAGAT-3′; whereas, the primers for Tenmo-SKR2 amplify fragment of 147 bp and were as follows Fw 5′-CCTTCCGTTATTTACAACAG-3′ and Rev 5′-CAGACACTTGAACAGACT-3′. The primers were synthetized by the Institute of Biochemistry and Biophysics of the Polish Academy of Science (Warsaw, Poland). PCR was performed in a 10-µL reaction volume containing 3.95 µL of DNase/RNase-free water, 1 µL of DreamTaq™ Green Buffer (Thermo-Fisher Scientific, USA), 1 µL of 2 mM dNTP, 1 µL of 10-µM forward primers, 1 µL of 10-µM reverse primers, 0.05 µL of DreamTaq™ DNA polymerase (Thermo-Fisher Scientific, USA) and 2 µL of cDNA. The obtained products were analyzed by electrophoresis using a 2% TAE agarose gel stained with ethidium bromide. The GeneRuler ™ 100 bp DNA Ladder (Fermentas, USA) was run on each gel. Photos of the agarose gels were taken using ChemiDoc™ Touch (Bio-Rad, USA). Minimum of 5 biological and 3 technical repeats were made. To confirm our results, the bands were sequenced with BigDye Terminator v3.1 on an ABI Prism 3130XL Analyzer (Applied Biosystems, Foster City, CA, USA) according to manufacturer's protocols by the Molecular Biology Techniques Laboratory (Faculty of Biology, Adam Mickiewicz University, Poznań) and compared with transcriptomic data. “No template control” and “no RT control” reactions were included in the analysis to ensure that there was no foreign DNA or genomic DNA contamination. In each analysis, RpS6 gene was used as an additional control.

### Peptides

Sulfated (pETSDDY(SO_3_H)GHLRFa) and nonsulfated (pETSDDYGHLRFa) sulfakinin (sSK and nSK, respectively), were obtained according to the procedures described previously by Słocińska et al. (2019).

### Injections and sample collections

To check the influence of SKs on ILPs or carbohydrate levels, each individual insect was injected with the hormone at dose 10 pmol or 20 pmol of sSK or nSK dissolved in 2 μL of Ringer solution (RS—274-mM NaCl, 19-mM KCl, 9-mM CaCl_2_) per injection. Control insects were injected with 2 μL of RS. After injections, all of the tested animal groups were kept at the same conditions as before injection. The peptide doses and the time point of sample collections were determined according to the previously prepared experiments. Hemolymph was collected 2 h and 24 h after hormone application. A sample of hemolymph per larva was collected from the first pair of legs after cutting the tarsus. Before experiments, insects were anesthetized by submerging them under water for 8 min.

### Qualitative and quantitative determination of free sugars in the hemolymph

The nature of the free sugars in the haemolymph of insects injected with SKs was analyzed by reverse-phase high-performance liquid chromatography (RP-HPLC). Hemolymph (4 µL) from larvae was collected to 600 µL of 70% ethanol and kept at 4 °C. The samples were centrifuged (5 min, 10,000 × *g*), and the supernatants were used for analysis of hemolymph components by RP-HPLC. Separations were performed using a Dionex Ultimate 3000 chromatographic system, comprising a dual-pump programmable solvent module and a Corona Charged Aerosol Detector (CAD). The supernatants were analyzed on an Asahipak NH2P-50 4E column (250 × 4.6 mm, Shodex, Japan). The samples were eluted with multi-step gradient of ACN concentration and flow rate as following: 0–5 min (86%, 1 mL/min), 5–10 min (83%, 1 mL/min), 10–20 min (81%, 1 mL/min), 20–38 min (81%, 1.4 mL/min) and 38–40 min (86%, 1 mL/min) at 38 °C. Trehalose, glucose, sorbitol, mannitol and inositol (Merck, Germany) were used as standards.

### Immunoenzymatic determination of the insulin-like peptides level in hemolymph

The level of the insulin-like peptides (ILPs) in insect hemolymph, after injection of SKs, was determined according to the method described by Chowański et al. (2018). Samples of hemolymph were collected from the insects 1 or 2 h after injection of 10 pmol or 20 pmol of sulfakinins. Hemolymph from 10 larvae was combined with 3 μL of 10% EDTA to obtain 35 μL of final sample. Next, the samples were shaken for 15 min (1400 rpm at 4 °C) on a thermomixer (Eppendorf) and stored at − 20 °C until analysis. Before measurements, the samples were centrifuged at 10,000 × *g* at 4 °C for 10 min. Analysis of ILPs level was prepared using an ELISA Insulin Kit (DRG International) targeted to human insulin, according to the protocol provided by the producer. Briefly, samples (25 μL) were added to the wells and incubated with Enzyme Conjugate for 30 min. After removal of the solution, the wells were washed 3 times with wash buffer, and Enzyme Complex was added. After 30 min of incubation and triple washing, the substrate solution was added, and the samples were incubated for 15 min. Finally, the reaction was stopped with the stop solution, and the absorbance was measured (*λ* = 450 nm) with a BioTek Spectrophotometer. The concentration of the ILPs was calculated based on a standard curve.

### Statistical analysis

All data are presented as the mean values ± SD of *n* number of replicates. For statistical comparison between the control and test group, Student’s *t* test was used. The statistical analyses were performed using Graph Pad Prism (version 5). Differences were considered statistically significant at *p ≤ 0.05, **p ≤ 0.01, and ***p ≤ 0.001.

## Results

### Analysis of *T. molitor* SKR1 and SKR2 sequences

**Fig. 1:**
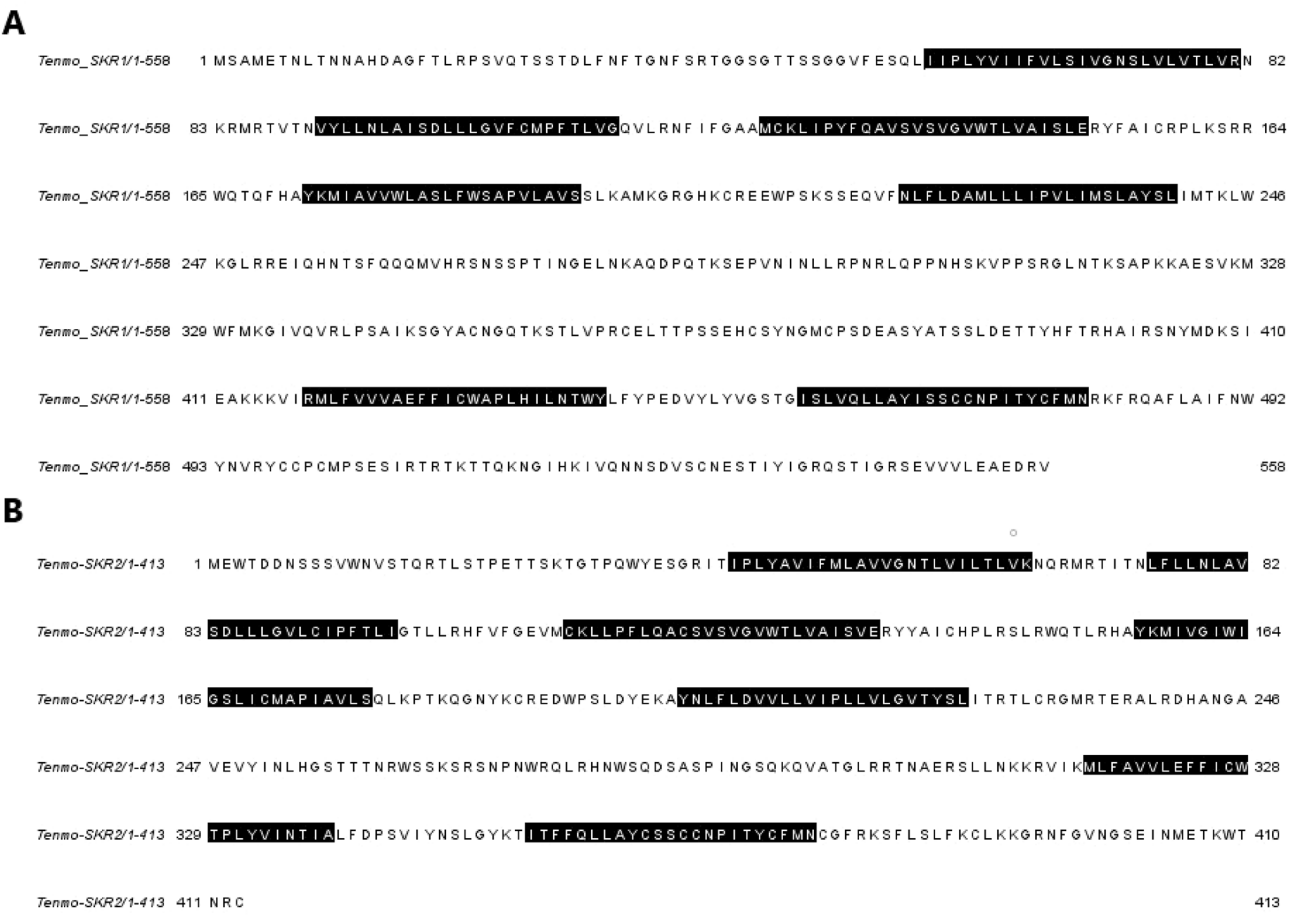
Amino acid sequences of SKR1 (**a**) and SKR2 (**b**) deduced from transcriptomic data of brain and retrocerebral complex of adult *T. molitor* beetle. Predicted transmembrane regions are highlighted in black

**Fig. 2:**
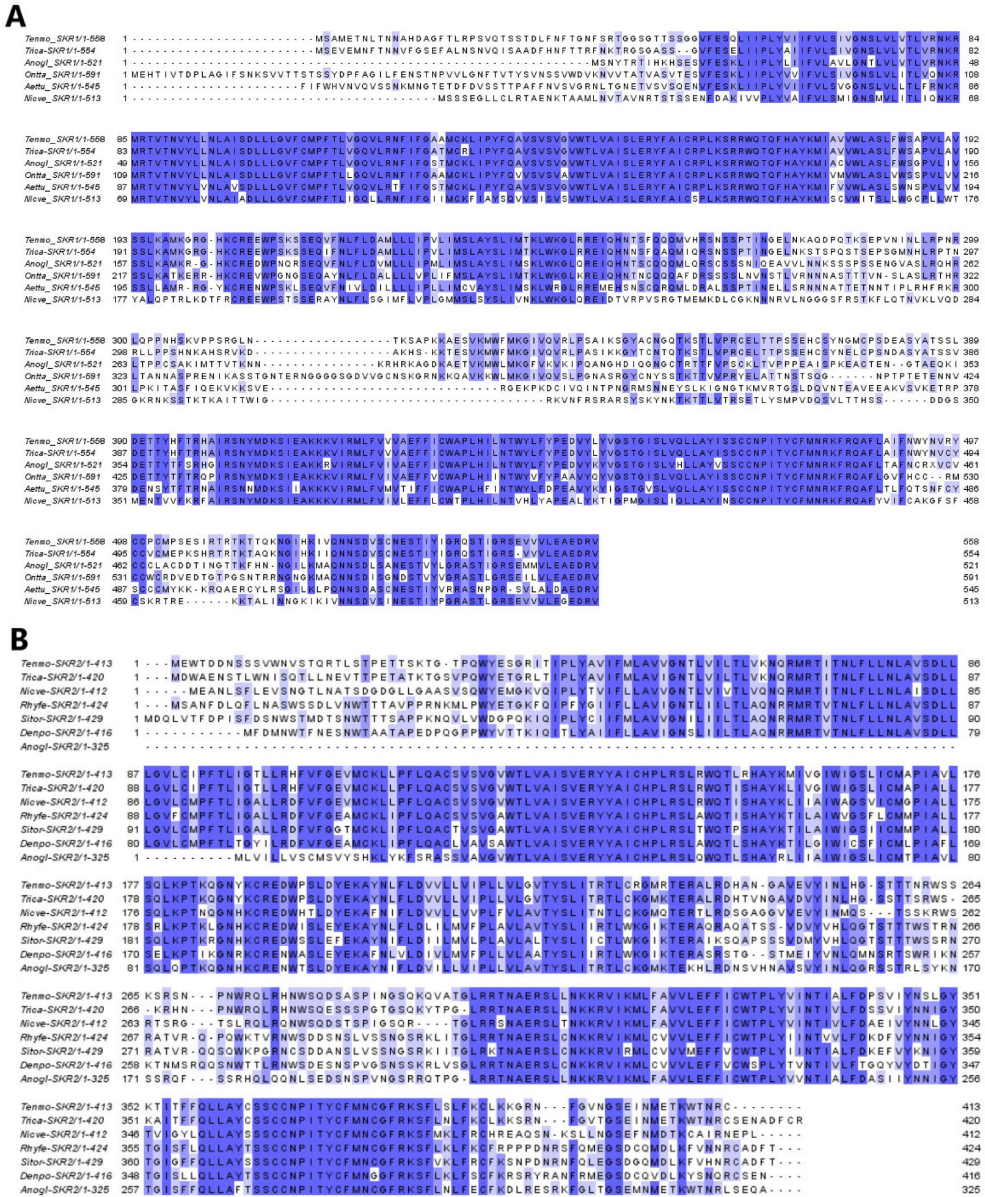
Sequence alignment of SKR1 (**a**) and SKR2 (**b**) amino acid sequences in chosen beetles. Identical and conserved amino acids across sequences are color coded in dark and light blue, respectively (color figure online)

Based on the BLAST search with local database, transcriptomic assembly of *T. molitor* brain and retrocerebral complex yielded in two open reading frames—1674 bp and 1233 bp which encode, respectively, a putative sulfakinin receptor 1 (SKR1) and sulfakinin receptor 2 (SKR2) (Fig. 1). Both, Tenmo-SKR1 and Tenmo-SKR2 display the seven transmembrane domains typical for GPCRs (Bass et al. 2014) with *N*-terminal ligand binding tail and C-terminal intracellular region (Fig. 1). Protein sequence alignment with other chosen beetles shows a very high degree of identity and similarity (Fig. 2). The highest similarity of Tenmo-SKR1 sequence was observed as expected with *T. castaneum* SKR1 (86%); whereas the lowest with *Nicrophorus vespilloides* (51%). Tenmo-SKR2 protein sequence was the most similar again to Trica-SKR2 (86%) and to *N. vespilloides* (69%). The lowest similarity was observed when compared to *Sitophilus oryzae* receptor (58%).

In both receptors, as expected, slightly higher variability was observed in N-terminal and C-terminal regions (Figs. 1, 2). Practically 100% identity was observed in sequences of transmembrane helices (Figs. 1, 2).

### Distribution of SKRs transcripts in different tissues of *T. molitor* larvae

**Fig. 3:**
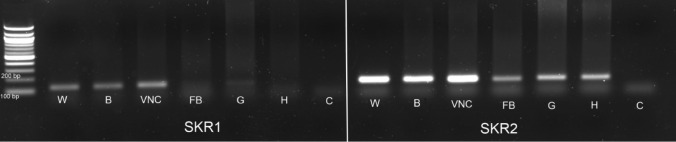
Distribution of SKR1 and SKR2 transcripts in different tissues of *T. molitor* *W* whole body, *B* brain, *VNC* ventral nerve cord, *FB* fat body, *G* gut, *H* hemolymph, *C* control (water)

To check whether the tested peptides affect analyzed tissues directly, we examined the Tenmo-SKR1 and Tenmo-SKR2 spatial distribution by RT-PCR. As a positive control, we used nervous tissues in which SKR was shown earlier to be present in *T. castaneum* (Yu et al. 2013; Zels et al. 2014). Analysis of SKRs transcripts distribution revealed that it is tissue specific, especially for SKR1. This receptor transcript has been found only in the nervous system (brain and ventral nerve cord) and gut (Fig. 3). The quantitative analysis was not performed; however, the band intensity indicates that the level of the transcript may vary between tissues. In the gut, it is very low; whereas, the most abundant is present in the ventral nerve cord (Fig. 3).

Contrary to SKR1, SKR2 transcript has been found in all tested tissues used for RNA isolation. It proves that SKR2 is present not only in the nervous system but as well in peripheral tissues such as fat body, gut and haemolymph of this beetle (Fig. 3). Band intensity indicates that the level of the transcript may vary between tested tissues and again is the highest in the nervous system and much lower in the peripheral tissues (Fig. 3).

### The effect of sulfakinins on the quality and quantity of the hemolymph sugars

**Fig. 4:**
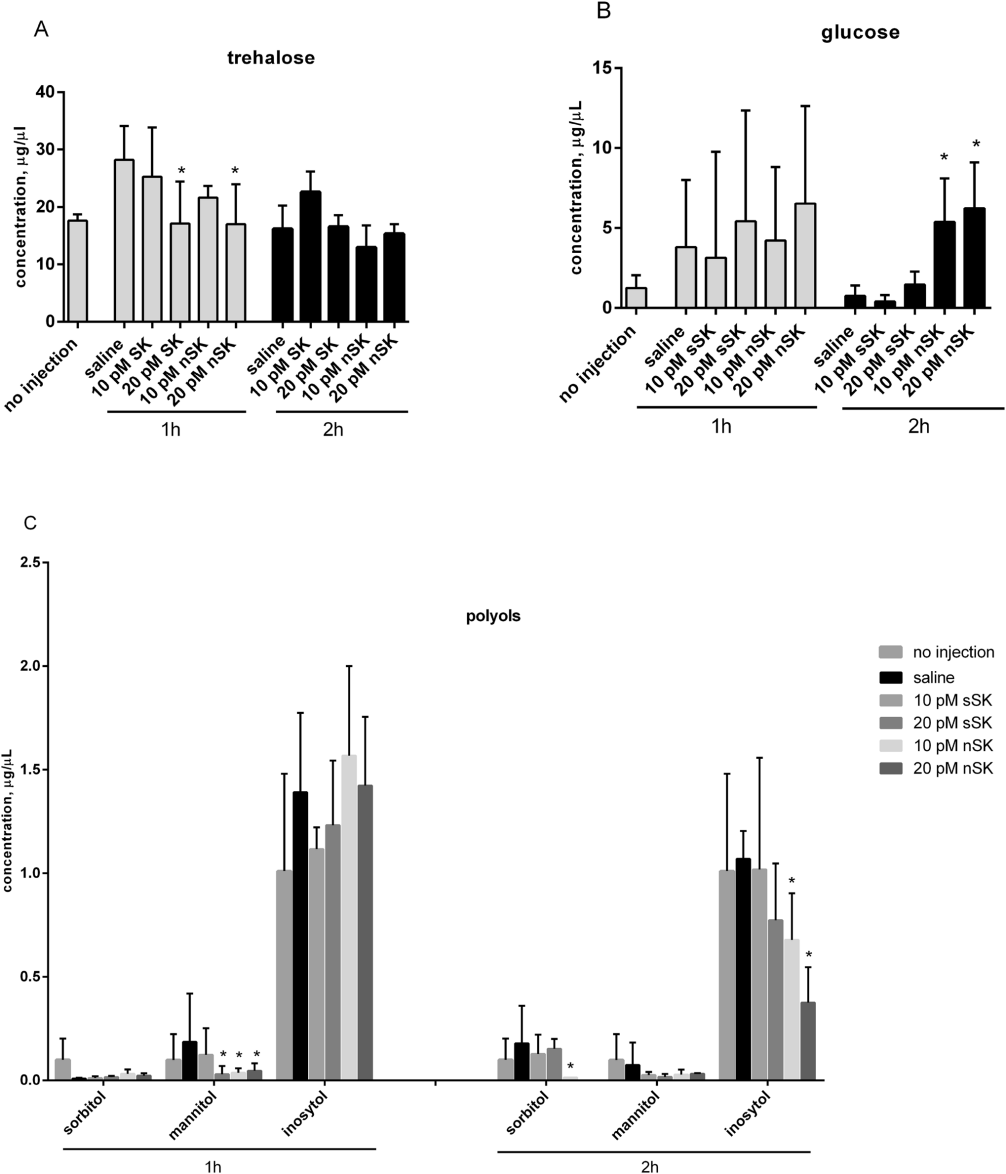
Changes in free sugar composition in the hemolymph of *T. molitor* larvae after sulfated (sSK) and nonsulfated (nSK) SKs administration. The control larvae were treated with saline. Hemolymph was collected 1 h and 2 h after saline or hormone injection. The data are shown as the mean ± SD from 6 to 10 repetitions. Statistically significant differences from the control values (saline) are indicated by asterisks (Student’s *t* test), where *p ≤ 0.05

The profile of free sugars in the hemolymph was determined using RP-HPLC method. The predominant sugar identified in hemolymph of *T. molitor* larvae was trehalose as was previously evidenced by Gäde and Rosiński (1990). This sugar was detected at concentration around 18 μg/μL of hemolymph. Relatively high concentration of glucose, of around 5 μg/μL (Fig. 4b) and sugar alcohols such as inositol—around 1.5 μg/μL, and mannitol—around 0.25 μg/μL, were detected (Fig. 4c) in *T. molitor* hemolymph; whereas, sucrose was present in trace amounts (not shown). The concentration of all identified sugars in control insects decreased in time and 2 h after injection was lower compared to the beginning of the experiment (Fig. 4). The concentration of trehalose dropped by around 35% after 1 h since application of 20 pmol of sSK as well as in case of nSK at the same doses. For the lower dose of hormones, the effect was definitely slighter thus statistically insignificant (Fig. 4a). 2 h after hormones treatment, the level of trehalose remained at similar level and did not differ significantly between control and tested group of insects (Fig. 4a). At the same time, the upward trend for glucose was observed; although after 1 h, the differences between control and experimental groups were not statistically significant. The strong hyperglycemic effect was noticed for nSK 2 h after peptides application. The level of glucose in this case increased almost sixfold (Fig. 4b). Sulfakinins changed the content of polyols, among them inositol occurred in major amount. The concentration of inositol, which equals around 1 μg/μL, decreased 2 h after hormones application to 0.6 and 0.4 μg/μL for 10 pmol of nSK and 20 pmol of nSK, respectively, compared to control insects. Not statistically important effect of SKs activity on inositol level was observed 1 h after hormone treatment (Fig. 4c). In this time variant, the effect of both SKs on mannitol level was observed; for nSK, the drop by around 60% was observed.

### The effect of sulfakinins on the level of insulin-like peptides in the haemolymph

**Fig. 5:**
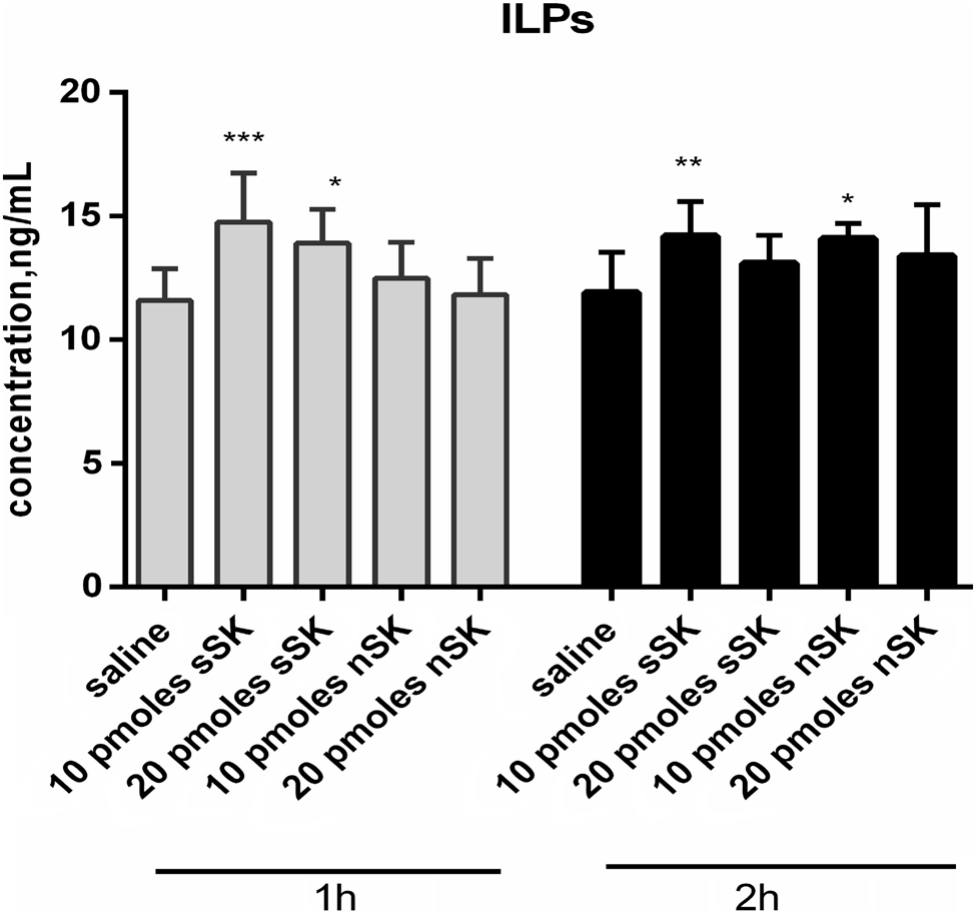
Concentration of ILPs (ng/mL) in the hemolymph of *T. molitor* larvae after sulfated (sSK) and nonsulfated (nSK) SKs administration. The control larvae were treated with saline. Hemolymph was collected 1 h and 2 h after saline or hormone injection. The data are shown as the mean ± SD from 6 to 10 repetitions. Statistically significant differences from the control values are indicated by asterisks (Student’s *t* test), where *p ≤ 0.05, **p ≤ 0.01, ***p ≤ 0.001

The level of ILPs in haemolymph of insects injected with saline was around 12 ng/mL and changed after sulfakinins application. Compared to control, 1 h after injection, sSK evoked stronger effect than nSK (Fig. 5). In this case, the increase in the ILPs level was observed, although the response was not concentration dependent. In contrary, 2 h after treatment, nSK induced more pronounced changes than after 1 h and increased ILPs concentration for 20% in statistically significant level (Fig. 5). However, the effect was lower than that observed for sSKs.

## Discussion

In this study, we reported localization and identification of sulfakinin receptors, SKR1 and SKR2, in larvae of *T. molitor* beetle. Moreover, we determined carbohydrate and ILPs level in hemolymph after sulfakinins application to show the involvement of these peptides in carbohydrate metabolism.

SKs signaling has been studied in variety of insect species, including flies (Kubiak et al. 2002), locusts (Zels et al. 2014), cockroaches (Wicher et al. 2007) or kissing bug (Al-Alkawi et al. 2017; Bloom et al. 2019). In most of these species, SK receptor (SKR) was identified and its protein sequence was analyzed. In beetles, the first SKR was identified in *T. castaneum* (Hauser et al. 2008). As for most of the neuropeptides, also SKRs belong to G-protein-coupled receptors with seven transmembrane helixes, N-terminal extracellular segment and the intracellular C-terminal tail responsible for interactions with G proteins (Hauser et al. 2008). Our results also confirm that two predicted receptors belong to these family. Both of them contain typical parts/segments as was shown in the alignments (Fig. 2).

Study on *T. castaneum* showed that two *skr* genes were expressed in the larval, pupal and adult insects with different levels in tested tissues (Yu and Smagghe 2014b; Yu et al. 2013). The highest expression was observed in the head throughout all developmental stages with prominent expression in larva stage for SKR1 and in pupa for SKR2 (Yu and Smagghe 2014b; Yu et al. 2013). Quantitative reverse transcriptase PCR shows that transcript levels of both receptors were primarily expressed in the central nervous system of adult stage of this beetle (Zels et al. 2014). In all other examined tissues, they occurred less abundantly. The transcript of SKR1 was found in fat body tissue, salivary gland, gut, ovaries and testes; whereas the transcript of SKR2 only in the brain and olfactory lobes (Zels et al. 2015). The analysis of distribution of SKRs in *T. molitor* larvae by RT-PCR reflects similarity to the presence of SKRs in adults of *T. castaneum* (Zels et al. 2014). Both SKR1 and SKR2 were identified mainly in brain and ventral nerve cord; however, the expression of SKR2 observed in *T. molitor* larvae was higher than SKR1, contrariwise as in *T. castaneum* beetle. The same effect was observed in *T. molitor* for SKRs transcripts in peripheral tissues such as fat body, gut or hemolymph. Their distribution profiles differ from that observed in adult insects of *T. castaneum* (Zels et al. 2014).

In *Rhodnius prolixus* instar, both sulfakinin receptors, Rhopr-SKR1 and Rhopr-SKR2, with higher than SKR2 expression of SKR1, has been identified not only in CNS, gut, fat body, male and female reproductive system, but also in heart and Malpighian tubules (Bloom et al. 2019). Our studies show that in *T. molitor* larvae, high expression of SKR2 in peripheral tissues was found in gut and hemolymph; whereas, the most abundant in *T. castaneum* adults was Trica-SKR1 in fat body tissue (Zels et al. 2014), and Rhop-SKR1 in male and female reproductive systems of *R. prolixus* 5th instar (Bloom et al. 2019). Thus, it may indicate that the distribution pattern of SKRs is dependent on the developmental stage and/or physiological state of insect; in turn, the higher amount of SKR2 may suggest for its more significant role in larva insects, at least in peripheral tissues. Yu and Smagghe (2014b) and Yu et al. (2013) showed that SKR2 in *T. castaneum* is involved in the regulation of feeding. In *R. prolixus* 5th instar, the knockdown of transcripts for Rhop-SKR1 and Rhop-SKR2 resulted in the increase of the mass of blood meal taken (Bloom et al. 2019). The presence of SKR2 transcript in gut and fat body of *T. molito*r is probably associated with myotropic and metabotropic activity of sulfakinins in these tissues. The role of SKs in gut biology and fat body metabolism was previously confirmed in several studies (Nichols 2007; Marciniak et al. 2011; Słocińska et al. 2016).

Interestingly, for the first time, we evidenced the presence of SKR in insect hemocytes. Their occurrence may suggest immunotropic functions of SKs, which are structurally and functionally similar to vertebrate cholecystokinin (CCK) and may play similar regulatory function as CCK in mammals (Guilloteau et al. 2006). Either sulfated or nonsulfated CCK exerted broad spectrum of effects on mammalian immune cells, including suppression of B cell costimulatory molecules, maturation of dendritic cells, murine macrophages, human neutrophils, and proinflammatory cytokines (Rehfeld 2017). For the first time, the presence of cholecystokinin receptor (CCKR) in immune system has been found in chicken immune cells, including monocytes (El-Kassas et al. 2016). Also, the engagement of neuropeptides such as adipokinetic hormone (AKH), insulin-like peptides (ILPs) or bursicon in insect immunity has been described (Urbański and Rosiński 2018). The high level of gene expression coding AKH receptors was observed in hemocytes of *Manduca sexta* caterpillars (Ziegler et al. 2011); whereas, the expression of genes coding insulin receptors (IR) was identified in granulocytes and oenocytes of *Aedes aegypti* (Castillo et al. 2011), which indicates the direct influence of these neuropeptide hormones on hemolymph cells.

In our study, we determined the level of trehalose and glucose in the larvae of *T. molitor*. After SKs administration, we observed decreasing tendency in concentration of trehalose, compared to control insects injected with saline. At the same time, glucose level increased, but statistically important elevation was observed only for nSK 2 h since injection (Fig. 4). The observed changes in sugar concentrations in *T. molitor* larvae under hormonal treatment confirm engagement of SKs in carbohydrate metabolism reported previously (Słocińska et al. 2016). Trehalose is a major blood sugar in insects, playing a crucial role as a source of energy. The hydrolysis of trehalose is under enzymatic control of trehalase leading to generation of glucose. The mechanism for regulation of trehalase is not fully understood, but is believed to be partially under hormonal control. Trehalase is the main link between trehalose metabolism and glucose transport in insects (Shukla et al. 2015). In our study, the lowering level of trehalose (especially 1 h since hormone application) and increasing level of glucose may indicate that trehalase activity is under SKs control. The opposite effect of nSK on carbohydrate levels was observed in *Z. atratus* beetle (Słocińska et al. 2015b), which may suggest the species-specific action of SK in insects. Interestingly, our results show that sulfation of tyrosine is not crucial for peptide effect on sugars level. Nonsulfated peptides act in even more efficient way regarding glucose or polyols. The opposite effects observed in related species, *T. molitor* and *T. castaneum* (Zels et al. 2014) may be a result of different experimental setup: injection of insects, as we did, and on the other hand, expression of SK in CHO cell line. In in vivo conditions, different signals from neuroendocrine system of insect may affect sulfakinin signaling as we previously reported in ligated larvae of *Z. atratus* beetle, e.g., adipokinetic hormone—AKH or other “head” factors (Słocińska et al. 2015a). Moreover, our previous study (Marciniak et al. 2011; Słocińska et al. 2016) and the present study indicate that the role of sSK and nSK can be related to developmental stage. We suggest the important role of nonsulfated peptides in maintaining of energy homeostasis in larval stage of insects.

Simultaneously, we determined the level of total insulin-like peptides (ILPs) in *T. molitor* larvae hemolymph after SKs application. The ELISA approach we applied does not allow to distinguish between different isoforms of ILPs, identified, between one and 38 in each species insects (Nässel and Vanden Broeck 2016). We can relate only their total amount to a function in carbohydrate metabolism based on the sensing of ILPs releasing to a circulating in the hemolymph glucose (Nässel and Vanden Broeck 2016). The increase in ILPs level was observed independent of the presence of sulfation moiety in the peptide structure. The more pronounced effect was observed for SKs after 1 h since application, the weaker, but still statistically significant increase of ILPs level was noted for nonsulfated peptide 2 h after its treatment. ILPs are important regulators of growth, metabolism, reproduction and lifespan, and have been identified in many insect species, but the most study has been conducted in fruitful *D. melanogaster* (Nässel and Vanden Broeck 2016). Secretion of ILPs by neurosecretory cells is controlled by different factors released from gut, and adipocytes or, indirectly by glucose-sensing cells producing adipokinetic hormone (AKH) (Nässel et al. 2015). ILPs and SKs are shown to be co-expressed in Drosophila brain in adults and larval insects. Lin et al. (2016) showed that ILPs and SKs interplay resulting in regulating of food intake in *T. castaneum*. The authors evidenced that reduced insulin signaling suppresses food intake leading to the decrease of weight gain and mortality during larval–pupal metamorphosis. ILPs role might depend on insect stage, e.g., larval stage which is characterized by extensive growing and developing of larval body. ILPs secretion is triggered not only by cells sensitive to increasing level of circulating glucose but also on stimuli of free fatty acids and amino acids. It might be the case for *T. molitor*, where the increased level of glucose (this study) and elevation of free fatty acids (FFA) after SKs titration (Słocińska et al. 2019) were observed. Similarly, as in *D. melanogaster*, release of ILPs from insulin-producing cells (IPC) depends on AKH signaling, such SKs may initiate secretion of ILPs from IPC. For AKH, such events may occur during nutritional stress to keep balance in carbohydrate homeostasis (Nassel et al. 2013). Thus, we may suppose a similar regulation of ILPs level by SKs to sustain energetic homeostasis required for insect growth and metabolism. One has to remember that apart from that, other neuropeptides such as neuropeptides F or short neuropeptides F might play a role in this interplay.

Summarizing, we have identified sulfakinin receptors, SKR1 and SKR2, in *T. molitor* beetle. The distribution of SKR2 in larval stage of *T. molitor* is more widespread than SKR1, and their transcripts have been found in peripheral tissues such as fat body, gut and hemolymph. The presence of SKR2 in insect hemocytes may indicate for the new, immunotropic activity of SKs. Moreover, we have demonstrated that these neuropeptide hormones regulate ILPs and carbohydrates level in hemolymph, and that sulfate moiety is not crucial for peptides activity. The present study confirms the role of SKs in management of energy homeostasis.

## References

[CR1] Adamski Z, Bufo SA, Chowański S, Falabella P, Lubawy J, Marciniak P, Pacholska-Bogalska J, Salvia R, Scrano L, Słocińska M, Spochacz M, Szymczak M, Urbański A, Walkowiak-Nowicka K, Rosiński G (2019). Beetles as model organisms in physiological, biomedical and environmental studies—a review. Front Physiol.

[CR2] Al-Alkawi H, Lange AB, Orchard I (2017). Cloning, localization, and physiological effects of sulfakinin in the kissing bug, *Rhodnius prolixus*. Peptides.

[CR3] Bass C, Katanski C, Maynard B, Zurro I, Mariane E, Matta M, Loi M, Melis V, Capponi V, Muroni P, Setzu M, Nichols R (2014). Conserved residues in RF-NH_2_ receptor models identify predicted contact sites in ligand–receptor binding. Peptides.

[CR4] Bloom M, Lange AB, Orchard I (2019). Identification, functional characterization, and pharmacological analysis of two sulfakinin receptors in the medically-important insect *Rhodnius prolixus*. Sci Rep.

[CR5] Castillo J, Brown MR, Strand MR (2011). Blood feeding and insulin-like peptide 3 stimulate proliferation of hemocytes in the mosquito *Aedes aegypti*. PLoS Pathog.

[CR6] Chen X, Ganetzky B (2012). A neuropeptide signaling pathway regulates synaptic growth in *Drosophila*. J Cell Biol.

[CR7] Chen X, Peterson J, Nachman RJ, Ganetzky B (2012). Drosulfakinin activates CCKLR-17D1 and promotes larval locomotion and escape response in *Drosophila*. Fly (Austin).

[CR8] Chowański S, Pacholska-Bogalska J, Rosiński G (2018). Cholinergic agonists and antagonists have an effect on the metabolism of the beetle *Tenebrio molitor*. Molecules.

[CR9] Downer KE, Haselton AT, Nachman RJ, Stoffolano JG (2007). Insect satiety: sulfakinin localization and the effect of drosulfakinin on protein and carbohydrate ingestion in the blow fly, *Phormia regina* (Diptera: Calliphoridae). J Insect Physiol.

[CR10] Duve H, Thorpe A, Scott AG, Johnsen AH, Rehfeld JF, Hines E, East PD (1995). The sulfakinins of the blowfly *Calliphora vomitoria*. Peptide isolation, gene cloning and expression studies. Eur J Biochem.

[CR11] El-Kassas S, Odemuyiwa S, Hajishengallis G, Connell TD, Nashar TO (2016). Expression and regulation of cholecystokinin receptor in the chicken's immune organs and cells. J Clin Cell Immunol.

[CR12] Gäde G, Rosiński G (1990). The primary structure of the hypertrehalosemic neuropeptide from tenebrionid beetles: a novel member of the AKH/RPCH family. Peptides.

[CR13] Guilloteau P, Le Meuth-Metzinger V, Morisset J, Zabielski R (2006). Gastrin, cholecystokinin and gastrointestinal tract functions in mammals. Nutr Res Rev.

[CR14] Harshini S, Nachman RJ, Sreekumar S (2002). In vitro release of digestive enzymes by FMRF amide related neuropeptides and analogues in the lepidopteran insect *Opisina arenosella* (Walk.). Peptides.

[CR15] Hauser F, Cazzamali G, Williamson M, Blenau W, Grimmelikhuijzen CJ (2006). A review of neurohormone GPCRs present in the fruitfly *Drosophila melanogaster* and the honey bee *Apis mellifera*. Prog Neurobiol.

[CR16] Hauser F, Cazzamali G, Williamson M, Park Y, Li B, Tanaka Y, Predel R, Neupert S, Schachtner J, Verleyen P, Grimmelikhuijzen CJ (2008). A genome-wide inventory of neurohormone GPCRs in the red flour beetle *Tribolium castaneum*. Front Neuroendocrinol.

[CR17] Kubiak TM, Larsen MJ, Burton KJ, Bannow CA, Martin RA, Zantello MR, Lowery DE (2002). Cloning and functional expression of the first *Drosophila melanogaster* sulfakinin receptor DSK-R1. Biochem Biophys Res Commun.

[CR18] Lin X, Yu N, Smagghe G (2016). Insulin receptor regulates food intake through sulfakinin signaling in the red flour beetle, *Tribolium castaneum*. Peptides.

[CR19] Maestro JL, Aguilar R, Pascual N, Valero ML, Piulachs MD, Andreu D, Navarro I, Belles X (2001). Screening of antifeedant activity in brain extracts led to the identification of sulfakinin as a satiety promoter in the German cockroach—Are arthropod sulfakinins homologous to vertebrate gastrins–cholecystokinins?. Eur J Biochem.

[CR20] Marciniak P, Kuczer M, Rosiński G (2011). New physiological activities of myosuppressin, sulfakinin and NVP-like peptide in *Zophobas atratus* beetle. J Comp Physiol B.

[CR21] Marone M, Mozzetti S, De Ritis D, Pierelli L, Scambia G (2001). Semiquantitative RT-PCR analysis to assess the expression levels of multiple transcripts from the same sample. Biol Proced Online.

[CR22] Nachman RJ, Giard W, Favrel P, Suresh T, Sreekumar S, Holman GM (1997). Insect myosuppressins and sulfakinins stimulate release of the digestive enzyme α-amylase in two invertebrates: the scallop *Pecten maximus* and insect *Rhynchophorus ferrugineus*. Ann N Y Acad Sci.

[CR23] Nassel DR, Kubrak OI, Liu Y, Luo J, Lushchak OV (2013). Factors that regulate insulin producing cells and their output in *Drosophila*. Front Physiol.

[CR24] Nässel DR, Liu Y, Luo J (2015). Insulin/IGF signaling and its regulation in *Drosophila*. Gen Comp Endocrinol.

[CR25] Nässel DR, Vanden Broeck J (2016). Insulin/IGF signaling in *Drosophila* and other insects: factors that regulate production, release and post-release action of the insulin-like peptides. Cell Mol Life Sci.

[CR26] Nichols R (2007). The first nonsulfated sulfakinin activity reported suggests nsDSK acts in gut biology. Peptides.

[CR27] Nichols R, Egle JP, Langan NR, Palmer GC (2008). The different effects of structurally related sulfakinins on *Drosophila melanogaster* odor preference and locomotion suggest involvement of distinct mechanisms. Peptides.

[CR28] Nichols R, Manoogian B, Walling E, Mispelon M (2009). Plasticity in the effects of sulfated and nonsulfated sulfakinin on heart contractions. Front Biosci (Landmark Ed).

[CR29] Nichols R, Schneuwly SA, Dixon JE (1988). Identification and characterization of a *Drosophila* homolog to the vertebrate neuropeptide cholecystokinin. J Biol Chem.

[CR30] Nugent T, Jones DT (2009). Transmembrane protein topology prediction using support vector machines. BMC Bioinform.

[CR31] Predel R, Brandt W, Kellner R, Rapus J, Nachman RJ, Gäde G (1999). Post-translational modifications of the insect sulfakinins: sulfation, pyroglutamate-formation and O-methylation of glutamic acid. Eur J Biochem.

[CR32] Rehfeld JF (2017). Cholecystokinin-from local gut hormone to ubiquitous messenger. Front Endocrinol (Lausanne).

[CR33] Schoofs L, Holman GM, Hayes TK, Nachman RJ, De Loof A, McCaffery AR, Wilson ID (1990). Isolation and identification of a sulfakinin-like peptide with sequence homology to vertebrate gastrin and cholecystokinin, from the brain of *Locusta migratoria*. Chromatography and isolation of insect hormones and prehormones.

[CR34] Shukla E, Thorat LJ, Nath BB, Gaikwad SM (2015). Insect trehalase: physiological significance and potential applications. Glycobiology.

[CR35] Słocińska M, Antos-Krzemińska N, Rosiński G, Jarmuszkiewicz W (2016). Nonsulfated sulfakinin changes metabolic parameters of insect fat body mitochondria. Arch Insect Biochem Physiol.

[CR36] Słocińska M, Czubak T, Marciniak P, Jarmuszkiewicz W, Rosiński G (2015). The activity of the nonsulfated sulfakinin Zopat-SK-1 in the neck-ligated larvae of the beetle *Zophobas atratus*. Peptides.

[CR37] Słocińska M, Kuczer M, Gołębiowski M (2019). Sulfakinin signalling influences fatty acid levels and composition in *Tenebrio molitor* beetle. Protein Pept Lett.

[CR38] Słocińska M, Marciniak P, Jarmuszkiewicz W, Rosiński G (2015). New metabolic activity of the nonsulfated sulfakinin Zopat-SK-1 in the insect fat body. Peptides.

[CR39] Söderberg JA, Carlsson MA, Nässel DR (2012). Insulin-producing cells in the *Drosophila* brain also express satiety-inducing cholecystokinin-like peptide, drosulfakinin. Front Endocrinol (Lausanne).

[CR40] Untergasser A, Cutcutache I, Koressaar T, Ye J, Faircloth BC, Remm M, Rozen SG (2012). Primer3—New capabilities and interfaces. Nucleic Acids Res.

[CR41] Urbański A, Rosiński G (2018). Role of neuropeptides in the regulation of the insect immune system—current knowledge and perspectives. Curr Protein Pept Sci.

[CR42] Veenstra JA (1989). Isolation and structure of two gastrin/CCK-like neuropeptides from the American cockroach homologous to the leucosulfakinins. Neuropeptides.

[CR43] Wicher D, Derst C, Gautier H, Lapied B, Heinemann SH, Agricola HJ (2007). The satiety signaling neuropeptide perisulfakinin inhibits the activity of central neurons promoting general activity. Front Cell Neurosci.

[CR44] Williams MJ, Goergen P, Rajendran J, Zheleznyakova G, Hagglund MG, Perland E, Bagchi S, Kalogeropoulou A, Khan Z, Fredriksson R, Schioth HB (2014). Obesity-linked homologues TfAP-2 and Twz establish meal frequency in *Drosophila melanogaster*. PLoS Genet.

[CR45] Yu N, Nachman RJ, Smagghe G (2013). Characterization of sulfakinin and sulfakinin receptor and their roles in food intake in the red flour beetle *Tribolium castaneum*. Gen Comp Endocrinol.

[CR46] Yu N, Smagghe G (2014). CCK(-like) and receptors: structure and phylogeny in a comparative perspective. Gen Comp Endocrinol.

[CR47] Yu N, Smagghe G (2014). Characterization of sulfakinin receptor 2 and its role in food intake in the red flour beetle, *Tribolium castaneum*. Peptides.

[CR48] Yu N, Zotti MJ, Scheys F, Braz AS, Penna PH, Nachman RJ, Smagghe G (2015). Flexibility and extracellular opening determine the interaction between ligands and insect sulfakinin receptors. Sci Rep.

[CR49] Zels S, Dillen S, Crabbe K, Spit J, Nachman RJ, Vanden Broeck J (2015). Sulfakinin is an important regulator of digestive processes in the migratory locust, *Locusta migratoria*. Insect Biochem Mol Biol.

[CR50] Zels S, Verlinden H, Dillen S, Vleugels R, Nachman RJ, Vanden Broeck J (2014). Signaling properties and pharmacological analysis of two sulfakinin receptors from the red flour beetle, *Tribolium castaneum*. PLoS ONE.

[CR51] Ziegler R, Isoe J, Moore W, Riehle MA, Wells MA (2011). The putative AKH receptor of the tobacco hornworm, *Manduca sexta*, and its expression. J Insect Sci.

